# Novel *TMEM127* Variant Associated to Bilateral Phaeochromocytoma with an Uncommon Clinical Presentation

**DOI:** 10.1155/2019/2502174

**Published:** 2019-10-27

**Authors:** Antía Fernández-Pombo, José M. Cameselle-Teijeiro, Jose A. Puñal-Rodríguez, Lourdes Loidi, Roberto Peinó-García, Paloma Cabanas-Rodríguez, Miguel Garrido-Pumar, Sandra Baleato-González, Enrique Flores-Ríos, David Araújo-Vilar

**Affiliations:** ^1^Division of Endocrinology, University Clinical Hospital of Santiago de Compostela, Spain; ^2^UETeM-Molecular Pathology Group, Department of Medicine, IDIS-CIMUS, University of Santiago de Compostela, Spain; ^3^Division of Pathology, University Clinical Hospital of Santiago de Compostela, Spain; ^4^Division of Surgery, University Clinical Hospital of Santiago de Compostela, Spain; ^5^Fundación Galega de Medicina Xenómica, Santiago de Compostela, Spain; ^6^Division of Pediatrics, University Clinical Hospital of Santiago de Compostela, Spain; ^7^Division of Nuclear Medicine, University Clinical Hospital of Santiago de Compostela, Spain; ^8^Division of Radiology, University Clinical Hospital of Santiago de Compostela, Spain

## Abstract

Phaeochromocytomas and paragangliomas are rare catecholamine-secreting tumours arising from the adrenal medulla or sympathetic paraganglia. It is known that 20–30% of all cases occur as a result of germline variants in several well known genes. The *TMEM127* gene was recently identified as a new phaeochromocytoma susceptibility gene. However, until a larger sample of cases is available, the prevalence, genotype-phenotype correlation, and a clear predominant biochemical pattern of *TMEM127*-related PCC, remain to be defined. We present a woman with the pathogenic variant c.86delG (p.Arg29Leu*f*s^∗^52) in the *TMEM127* gene, which has not been previously reported, associated to a bilateral phaeochromocytoma, with an uncommon initial clinical presentation and a biochemical profile that is distinctly adrenergic. Her two young children carry the same variant and are, at present, disease-free. Physicians should be aware that phaeochromocytoma can manifest in an atypical manner, as with episodic hypotension, mainly if the symptoms have no obvious aetiology and they worsen over time. This case also supports the presence of a predominant adrenaline secreting pattern in *TMEM127*-positive tumours, as well as the need to consider multigene panel testing in patients with bilateral phaeochromocytomas.

## 1. Introduction

Phaeochromocytomas (PCC) and paragangliomas are rare neural crest-derived tumours found respectively in the adrenal medulla or sympathetic paraganglia [1]. These tumours are usually benign and sporadic. However, 20–30% of all cases are part of well characterized hereditary tumour syndromes [2], such as von Hippel-Lindau disease, Multiple Endocrine Neoplasia type 2 and Neurofibromatosis type 1, which occur as a result of germline variants in *VHL, RET, NF1*, and *SDH* (subunits A, B, C, D and AF2) genes [3]. In addition, in 2010–2011, the *TMEM127* and *MAX* genes were also identified as new PCC susceptibility genes [4, 5]. The *TMEM127* gene encodes a transmembrane protein that is linked to the regulation of the mTOR signaling complex. The majority of the variants described so far are associated with truncation of the protein, suggesting that they result in loss of *TMEM127* function [4]. Here, we present a family with a novel pathogenic variant in the *TMEM127* gene, associated to a bilateral PCC.

## 2. Case Presentation

A 33-year-old woman first consulted at the Division of Cardiology because of presyncopal symptoms, which started just after the delivery of her first child, with acral pallor, associated with pain, tremor and weakness in the lower limbs. In addition, she presented gastrointestinal symptoms with epigastric pain and nausea, accompanied by slow palpitations and significant orthostatic hypotension. During one of the episodes an electrocardiogram showed a nodal rhythm with a ventricular rate of 50. The Holter revealed a clear relation between the symptomatology and these episodes of nodal rhythm. Seven years later, after a routine blood test she showed an impaired fasting glucose of 110 mg/dl and was referred to the Division of Endocrinology. During a thorough anamnesis, the patient reported worsening of the symptoms and an increase in their frequency, with the appearance of pulsating headaches, dizziness, tinnitus and high blood pressure (up to 225/117 mmHg) followed by hypotension (63/36 mmHg on average). As far as her medical history is concerned, she had undergone a thyroid lobectomy with isthmusectomy, with a histological finding of a nodular hyperplasia. There was no family history of endocrine disease. However, her mother had hypertension and her father passed away due to a brainstem stroke. She has two healthy children.

**Figure 1:**
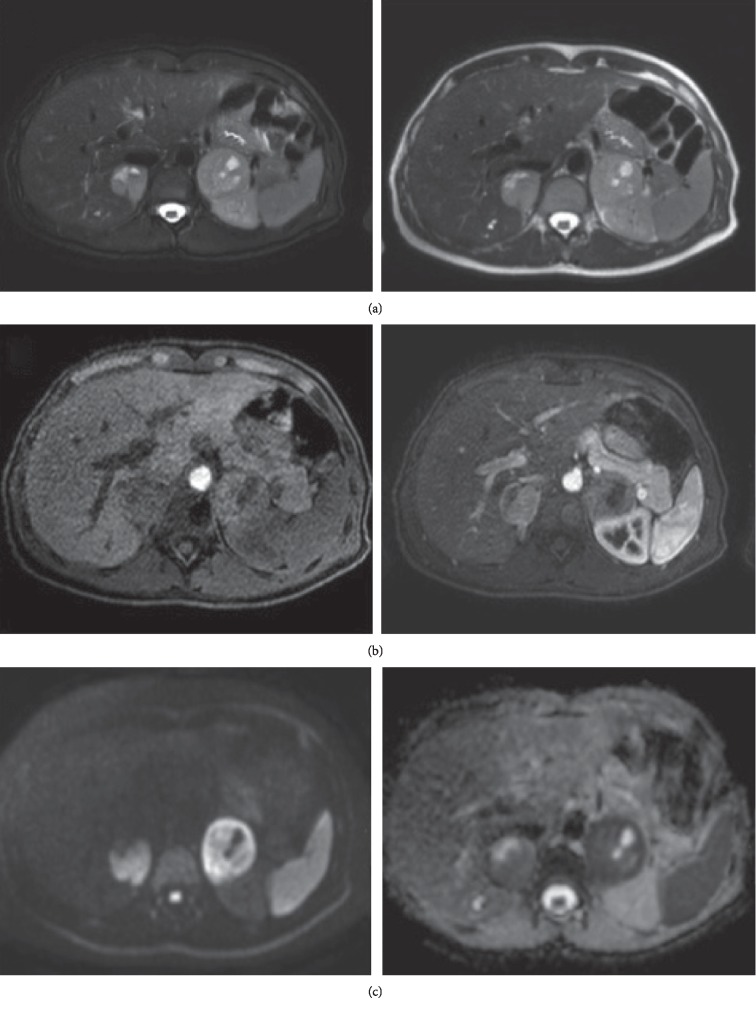
Abdominal MRI scan showing bilateral phaeochromocytoma. (a) axial TSE-T2W images with and without fat suppression; (b) axial GRE-T1W images prior to and 35 s after contrast injection; (c) DWI and ADC map showing restricted diffusion (ADC = 0.6 × 10^−3^ mm^2^/s).

**Figure 2:**
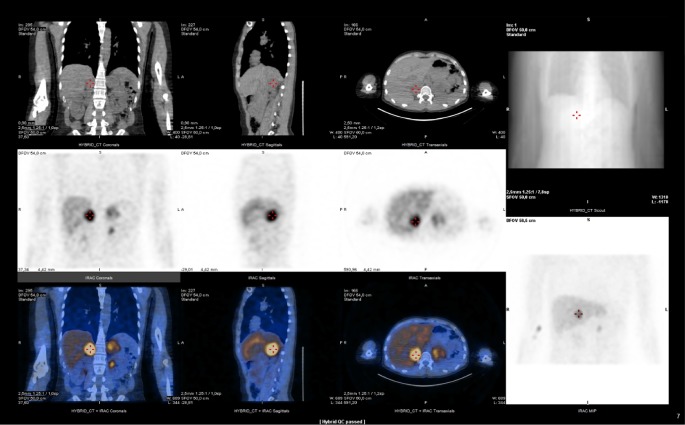
^123^I-MIBG SPECT/CT showing increased uptake of the tracer in both adrenal glands, with right predominance.

With the clinical suspicion of PCC, an analytical study was requested, which showed: urine adrenalin 367 mcg/24 h (<20 mcg/24 h), urine noradrenalin 81 mcg/24 h (15–80 mcg/24 h), urine metanephrine 2490 mcg/24 h (25–312 mcg/24 h) and urine normetanephrine 816 mcg/24 h (60–757 mcg/24 h). Magnetic resonance imaging (MRI) (Figure 1) revealed bilateral adrenal masses, 37 mm right and 49 mm left. It showed a hyperintense signal of both adrenal glands on fat-suppressed T2-weighted, without the presence of fat. After gadolinium administration these masses enhanced avidly and heterogeneously with regions of no enhancement due to cystic changes. Restricted diffusion (*b* = 800) of hypercellular areas was well demonstrated by ADC map as areas of low intensity signal. In oppossite, cystic components demonstrated high signal. ^123^I-MIBG SPECT/CT (Figure 2) showed increased uptake foci in both adrenal glands, with right predominance, compatible with the diagnosis of bilateral PCC. No extraadrenal tumour localization was detected.

**Figure 3:**
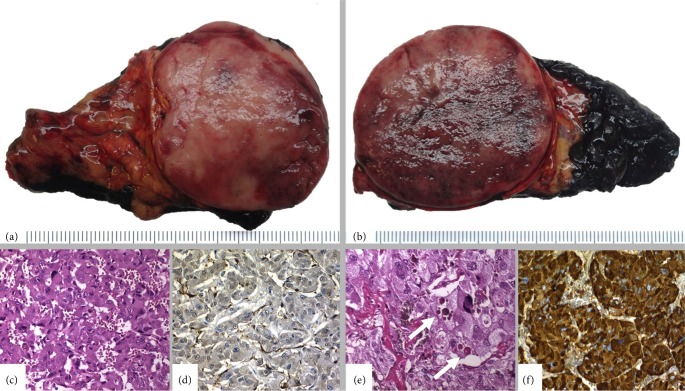
Bilateral phaeochromocytoma. Both right (a, c, d) and left (b, e, f) adrenal tumours were similar in the macroscopic and microscopic appearance. Microscopically they showed the typical nested pattern (c, e), moderate pleomorphism and occasional intracytoplasmic hyaline globules (arrows), but no mitotic activity. S100 sustentacular cells (d) and positivity for chromogranin A (f) were demonstrated. The Ki-67 was less than 2%.

The patient underwent a laparoscopic bilateral adrenalectomy. The bilateral tumours were circumscribed but unencapsulated, and partially surrounded by a bright-yellow ring of normal adrenal cortical cells. Both tumours also showed a fleshy, tan cut surface, with haemorrhage areas, measuring 4.5 cm and 5.4 cm maximum diameter in the right and left glands respectively (Figure 3).

Genetic testing was carried out. The patient presented the pathogenic variant NM_017849.3: c.86delG (p.Arg29Leufs^∗^52) in heterozygosis in the *TMEM127* gene. This variant has not been reported in available databases (i.e., dbSNP146, 1000 Genomes, ExAC and GnomAD). This consists of the deletion of one nucleotide in exon 2 of *TMEM127* which alters the reading pattern and leads to the appearance of a premature stop codon. Given that this premature stop codon is 7 nucleotides upstream the next exon-junction complex, it is unlikely that this new transcript is down regulated by nonsense-mediated decay [6]. This result suggests a susceptibility to PCC with an autosomal dominant inheritance pattern (OMIM #171300). Given these findings, a genetic study was performed on her children, aged 9 and 6, who were proved to be carriers of the same variant in the *TMEM127* gene. Plasma and urine catecholamines and metanephrines and total body MRI showed normal results in both children. However, given the risk of developing PCC or other tumours (renal carcinoma) [7], they will require surveillance. In addition, her mother tested negative for the variant.

One year after the adrenalectomy the patient is asymptomatic, with normal metanephrine excretion. As expected, she has primary adrenal insufficiency and is being treated with hydrocortisone.

## 3. Discussion

To the best of our knowledge, this is the first report of a Spanish patient showing that a *TMEM127* variant plays a pathological role in bilateral PCC. Furthermore, this c.86delG variant has not been previously reported.

Patients carrying *TMEM127* pathogenic variants have a similar age of tumour diagnosis as those who have sporadic lesions (41.5 years vs. 45 years); one-third have bilateral disease; the tumours are usually benign; the occurrence of catecholamine excess with symptoms precede PCC diagnosis by several years, and only 25% have a family history of PCC [4, 8, 9]. In this case, in spite of its paroxysmal occurrence, indicative of PCC, the atypical clinical scenario caused a delay in the diagnosis. It is necessary to consider that the presence of sinus node dysfunction may be an initial manifestation of PCC [10]. Physicians must also be aware that it may likewise present with hypotension (excessive stimulation of beta adrenoceptors by elevated levels of epinephrine), postural hypotension or alternating episodes of high and low blood pressure [11]. However, a clear genotype-phenotype correlation is unlikely to become evident until a larger sample of these families is published. It has also been suggested that these patients can likewise develop cathecholamine-mediated arterial vasospasm, leading to cerebral ischemia [12]. Thus, in this case, it is not unreasonable to think that either the patient has a de novo variant or the variant comes from the deceased father, who passed away due to a brainstem stroke.

Regarding the biochemical profile, while in an international cohort of 990 patients with PCC, of whom 2% presented pathogenic *TMEM127* germline variants, no preference of adrenaline or noradrenaline secretion was reported [8], three other studies have reported a predominant adrenaline secreting pattern [7, 13, 14], which we have also described in this case, further raising the suspicion of an etiology distinct from *VHL* or *SDHB* germline variants [15].

At present, the exact prevalence of *TMEM127*-related PCC is still unknown and will need to be defined by the *TMEM127* genotyping of further large series. Moreover, due to the large number of genes responsible for the development of PCC, genetic testing remains a diagnostic challenge. In addition, it has been reported that 3% of apparently sporadic PCC were caused by *TMEM127* gene variants [4]. Taking into account the significant implications for patients and their family members, the identification of a possible underlying genetic variant is important. Thus, advances in DNA sequencing technologies that make possible simultaneous testing for multiple genes should be used.

This case supports the need to add *TMEM127* to the multigene panel testing in patients with bilateral PCC with a predominant adrenaline secreting pattern, even if there is low suspicion of a hereditary condition.

## Data Availability

The data that support the findings of this study are openly available in at https://databases.lovd.nl/shared/variants/TMEM127/unique, reference number #00234343.
